# Paradoxical Worsening of Pulmonary Hypertension Following Closure of Arteriovenous Fistula: A Case Report and Literature Review

**DOI:** 10.7759/cureus.50064

**Published:** 2023-12-06

**Authors:** Abdul Muhsen Z Abdeen, Zakaria Alagha, Caleb Clark, Amro Al-Astal

**Affiliations:** 1 Internal Medicine, Marshall University Joan C. Edwards School of Medicine, Huntington, USA; 2 Internal Medicine/Pulmonology, Marshall University Joan C. Edwards School of Medicine, Huntington, USA

**Keywords:** pulmonary hypertension, end-stage renal disease, heart failure with preserved ejection fraction, carbon monoxide diffusion capacity, arteriovenous fistulae

## Abstract

This case report presents the atypical instance of a 59-year-old female patient with end-stage renal disease (ESRD) who was initially referred to the pulmonary clinic for evaluation due to a low diffusing capacity of the lung for carbon monoxide (DLCO). Pulmonary hypertension (PH) was suspected, and a subsequent right heart catheterization (RHC) confirmed PH attributed to group 5 PH, leading to the decision to close the unused arteriovenous fistula (AVF) to manage PH. Unexpectedly, a follow-up RHC showed a worsening of PH with elevated pulmonary capillary wedge pressure (PCWP), revealing an additional component of post-capillary group 2 PH. This case emphasizes the significance of recognizing a low DLCO as a potential trigger for PH assessment, especially in patients with comorbidities like ESRD. Furthermore, it highlights the unusual yet critical occurrence of PH exacerbation following AVF closure.

## Introduction

Pulmonary hypertension (PH) is a multifaceted condition with various etiological classifications. It impacts individuals of all age groups worldwide, with an estimated prevalence of approximately 1%. This condition carries a significant risk to life, demonstrated by an age-adjusted mortality rate of 7.9/100,000 individuals [1-3]. Patients with PH secondary to end-stage renal disease (ESRD) fall under group 5, which is influenced by factors related to chronic kidney disease [4]. In this case report, we present an atypical case of a 59-year-old female patient with ESRD initially referred to the pulmonary clinic due to a low single-breath carbon monoxide diffusing capacity (DLCO), diagnosed with PH via right heart catheterization (RHC), and interestingly, PH unexpectedly worsened following the closure of the arteriovenous fistula (AVF). A positive response to a fluid challenge indicated the presence of previously undiagnosed cardiac dysfunction as a potential underlying cause.

## Case presentation

A 59-year-old woman with a history of ESRD undergoing hemodialysis via a recently placed permcath, along with type 2 diabetes mellitus and hypertension, was referred to the pulmonology clinic for evaluation due to unexplained low DLCO during preoperative assessments in preparation for renal transplantation. The patient's surgical history includes an AVF that has not been utilized and the recent placement of a permcath in anticipation of the transplant. The patient had a lifelong history of non-smoking and no previous lung conditions. Her only complaint was progressive exertional dyspnea.

During the examination, the patient was afebrile, with a blood pressure of 160/90 mmHg, a pulse rate of 98 beats/min, a respiratory rate of 20 breaths/min, and an oxygen saturation of 96% on room air. Physical examination revealed decreased breath sounds on the right lung base side, particularly in the lower right hemithorax, with reduced percussion resonance. No murmurs were auscultated, and the remainder of the physical examination yielded unremarkable findings.

Further evaluation was conducted, involving pulmonary function tests that showed no evidence of obstructive or restrictive lung disease but unexpectedly revealed a low corrected DLCO of 26%. High-resolution CT chest scan findings indicated no signs of interstitial lung disease but did show the presence of pleural effusion on the right side. A ventilation/perfusion scan ruled out pulmonary embolism. Apart from the dilated left atrium, the transthoracic echocardiography (TTE) study did not reveal any other notable findings, and there were no echocardiographic signs suggesting the presence of PH or intracardiac shunt. Apart from elevated creatinine at 4.79 mg/dl, laboratory workup was unremarkable. Additionally, an autoimmune work-up yielded negative results. Additionally, the flow rate through AVF was measured at 3516 ml/min by color Doppler.

Given the unexplained low DLCO, a RHC was performed to evaluate possible PH. The RHC revealed elevated mean pulmonary arterial pressure and pulmonary capillary wedge pressure (PCWP) (Table 1). Based on these findings, a diagnosis of group 5 PH, likely associated with ESRD and the AVF, was made. Consequently, a decision was made to close the unutilized AVF, with a plan to conduct a repeat RHC 12 weeks after closure to reassess pulmonary hemodynamics. The follow-up RHC (Table 1) revealed elevated pulmonary pressures and a rise of PCWP from 15 to 19 mmHg after a 500 cc saline fluid challenge. V wave was higher than the A wave and went up further after the fluid bolus. The vasoreactivity test was negative. Additionally, the previously noted high mixed venous saturation, high cardiac output, and low pulmonary vascular resistance (PVR) resolved after the shunt was closed. These findings uncovered an additional component contributing to PH. The positive response to the fluid challenge strongly suggested the presence of additional postcapillary PH, likely falling into the category of group 2 PH. This was attributed to previously undiagnosed heart failure with preserved ejection fraction (HFpEF).

**Table 1:** Hemodynamic measurements before and after AVF closure on right heart catheterization AVF: Arteriovenous fistula

Measurements	Prior to AVF Closure	After AVF Closure
Pulmonary artery pressure (mmHg) (systolic/diastolic/mean)	55/18/30	64/17/35
Pulmonary capillary wedge pressure (mmHg)	20	15
Pulmonary capillary wedge pressure( mmHg) following fluid challenge	N/A	19
Cardiac index by thermodilution method (L/min/m^2^)	4.48	3.56
Pulmonary vascular resistance (Wood unit)	1.60	3.86
Systemic vascular resistance (dynes-sec/cm^5^)	1945	2854
Right atrial saturation (%)	86	70
Right ventricular saturation (%)	80	68

The subsequent TTE displayed a three-dimensional (3D) volume ejection fraction (EF) of 51%. The left atrial volume index (LAVI) measured 42.8 ml/m2, while the left ventricular mass index (LVMI) measured 142 gm/m2. Additionally, a moderately dilated left atrium (4.5 cm) was observed, alongside an E/A ratio of 1.0 and a mitral valve deceleration time of 200 milliseconds accompanied by trans-mitral spectral flow patterns, suggestive of a pseudo-normalization pattern. These findings are consistent with HFpEF.

After these findings, the patient's treatment plan was adjusted. She was started on sildenafil to address PH. Additionally, guideline-directed medical therapy for HFpEF was initiated. This comprehensive approach aimed to effectively manage both her PH and heart failure.

The patient responded well to the tailored treatment plan and her symptoms improved and she eventually became eligible for renal transplantation.

## Discussion

PH is a hemodynamic condition defined by an elevated mean pulmonary artery pressure (mPAP) of 20 mmHg or higher, as per the latest guidelines from the World Symposia on Pulmonary Hypertension Association. PH can be categorized into five distinct sub-groups, each presenting with its unique etiological factors and underlying pathophysiological mechanisms [5].

While various diagnostic tests aid in PH diagnosis, a high degree of suspicion is essential, especially when faced with unexplained symptoms like shortness of breath. Although an echocardiogram can assist in assessing the likelihood of PH, suspicion may persist even when echocardiographic results indicate a low probability, as exemplified by our patient who did not exhibit any signs suggestive of PH. Consequently, less-utilized diagnostic tools like DLCO become paramount in evaluating PH probability [3].

In systemic sclerosis patients, a predicted DLCO < 60% strongly associates with PH, especially in mildly symptomatic cases. Additionally, when DLCO/alveolar volume falls below 60% of predicted values, it indicates eventual PH development within 36 months. DLCO is a valuable prognostic and follow-up marker, integrated into the REVEAL (Registry to Evaluate Early And Long-term PAH Disease Management) score of 1.0 for predicting survival in PH patients. A DLCO value < 50% predicts pulmonary vascular disease. Despite its exclusion from recent PH diagnosis guidelines, DLCO remains a crucial diagnostic tool, emphasizing its significance in diagnosing PH, with a threshold of ≤40% in the REVEAL 2.0 version [6].

Therefore, DLCO is a well-established pulmonary function test used to evaluate patients with suspected PH. In our case, when our patient exhibited significantly reduced DLCO values, it raised concerns about potential PH. It was suggested that the actual DLCO measurement might be even lower due to the left-to-right shunt through the AVF and the high cardiac output state, prompting us to conduct further investigation through an RHC.

This case provided additional insights when closing AVF resulted unexpectedly in a paradoxical worsening of PH, raising important considerations. To illustrate, PH has been documented in as many as 33% of ESRD patients undergoing hemodialysis. The emergence of PH in renal patients is believed to be triggered by factors such as uremic endothelial dysfunction, chronic inflammation, and the formation of AVF. It is suggested that the creation of AVF may have a significant impact on the exacerbation of PH due to various factors. AVFs can disrupt preload and afterload dynamics, impacting cardiac workload and resistance, occasionally resulting in a high cardiac output state. AVFs in high-output heart failure create a low-resistance shunt, lowering systemic vascular resistance and increasing venous return. Consequently, compensatory mechanisms are triggered, leading to increased cardiac workload and left ventricular hypertrophy, contributing to high-output heart failure. Recent research has revealed a significant relationship between flow rates and the development of PH. In particular, individuals who developed PH have a substantially higher average flow rate of 2750 mL/min, while those without PH have a lower average flow rate of 1322 mL/min. Notably, in our specific case, the patient's flow rate measured a considerably elevated 3516 mL/min, which may provide additional insight into the role of flow rates in the development of PH in this particular patient [7-9].

Interestingly, the compression or ligation of the fistula has been observed to result in a significant decrease or even normalization of pulmonary pressures. Additionally, closing AVFs is a common approach to managing high-output states, typically resulting in either improvement or minimal changes in PH [7,10]. In a study involving patients with ESRD, heart failure, and PH, all treated patients exhibited improved symptoms, decreased cardiac output, and reduced pulmonary artery pressures [11]. In our patient, the primary intention behind closing the AVF was to manage PH. However, during a subsequent RHC, unexpected results were observed. These included paradoxical increases in mPAP, and instead of a decrease in PCWP, it actually increased to 19 mmHg following a fluid challenge. These findings strongly suggest the presence of another underlying pathology contributing to PH, distinct from group 5 PH. Specifically, it appears to be indicative of group 2 PH due to underlying undiagnosed HFpEF.

In patients with a PCWP of ⩽15 mmHg, a fluid challenge can be employed to uncover potential left ventricular diastolic dysfunction. The majority of current research focuses on identifying HFpEF by observing an elevation in PCWP. Typically, it is generally acknowledged that administering a rapid infusion of approximately 500 mL of saline over a span of 5-10 minutes is an effective method for detecting an abnormal increase in PCWP, which can reach ⩾18 mmHg and is indicative of HFpEF [12]. In light of this, we hypothesize that in cases involving normal hearts, as previously discussed, closing the AVFs would typically result in a decrease in pulmonary pressures due to the relief of the high-output state and the restoration of pulmonary flow to normal levels. However, in our case, when the AVF was closed, the subsequent increase in afterload raised myocardial workload, leading to a further elevation of pulmonary artery pressures, particularly due to the persistence of the post-capillary component.

To illustrate, chronic kidney disease (CKD) is a recognized cardiovascular risk factor, associated with many cardiovascular complications due to the intricate shared pathophysiological mechanisms between the cardiovascular system and the kidneys. This complicates the diagnosis of PH in CKD patients. Limited research has focused on validating PH through RHC in CKD patients. However, a noteworthy retrospective study was carried out at a single center, involving 1873 CKD patients who underwent RHC which may also explain these findings. The study showed that CKD is independently linked to PH with post-capillary PH, accounting for 76% of cases [13,14]. Additionally, some studies have indicated that the prevalence of PH and heart failure in patients undergoing hemodialysis may be as high as 42% [15].

Considering the patient's diagnosis of combined group 2 and group 5 PH due to heart failure and ESRD, respectively, an optimal management approach involves optimizing heart failure treatment and dialysis until renal transplantation becomes feasible. It's essential to exercise caution when exploring treatment options for PH in the context of heart failure, as endothelin receptor inhibitors and prostaglandin agonists are generally avoided in such cases [16-18]. In this specific case, sildenafil, a phosphodiesterase-5 inhibitor, was chosen as a therapeutic option for the PH component. This choice is supported by multiple studies demonstrating its efficacy in improving exercise capacity and pulmonary hemodynamics [19].

Despite the comprehensive assessment presented in this case report, we acknowledge certain limitations. Our analysis is based on a single patient, limiting the generalizability of our findings. Furthermore, the underlying mechanisms leading to the paradoxical worsening of pulmonary hypertension post-AVF closure require further elucidation through additional research. Future studies involving larger cohorts of ESRD patients and rigorous hemodynamic monitoring are warranted to provide a more comprehensive understanding of this phenomenon and to guide therapeutic interventions. Moreover, future research could explore the impact of PH treatment on DLCO and its potential prognostic relevance.

## Conclusions

Maintaining a high level of clinical suspicion and carefully evaluating DLCO values is crucial when assessing PH, as demonstrated in our patient whose reduced DLCO values served as the catalyst for diagnosing PH. It also emphasizes that worsening PH post-AVF closure in an ESRD patient, with a positive response to fluid challenge, suggests an underlying, previously undiagnosed HFpEF.
